# Adaptive Spoofing Suppression Algorithm for GNSS Based on Multiple Antennas Array

**DOI:** 10.3390/s20041115

**Published:** 2020-02-18

**Authors:** Guangwei Fan, Xingli Gan, Baoguo Yu, Qiang Rong, Chuanzhen Sheng

**Affiliations:** 1 State Key Laboratory of Satellite Navigation System and Equipment Technology, Shijiazhuang 050081, China; fgweihb@163.com (G.F.); yubg@sina.cn (B.Y.); rong_54@163.com (Q.R.); shengchuanzhen@163.com (C.S.); 2 The 54th Research Institute of China Electronics Technology Group Corporation, Shijiazhuang 050081, China

**Keywords:** satellite navigation, spoofing, suppression, cross-correlation, adaptive nullification

## Abstract

The signals of navigation satellites are easily affected by spoofing interference, causing the wrong position, speed or Universal Time Coordinate of the receiver to be calculated. Traditional detection and suppression algorithms are used only to eliminate the spoofing signals, which may lead to an insufficient number of satellites for positioning. An adaptive spoofing suppression algorithm (ASSA) based on a multiple antenna array is proposed in this study. The ASSA can use the cross-correlation gain of multiple antenna array to adaptively generate nulling and realize the simultaneous suppression of multiple spoofing signals. Moreover, ASSA does not need to capture and track spoofing separately, thus reducing the complexity of implementation and calculation. Experiments were conducted to verify the proposed system under different conditions, and the results show that ASSA can suppress multiple spoofings with little impact on positioning performance. Under the condition of spoofing, ASSAs were (2.22 m, 2.41 m, 4.43 m) in the static test and (2.27 m, 2.43 m, 4.64 m) in the kinematic test, which are good positioning performances for both. In addition, the ASSA is applied before capturing signals, which is beneficial to identifying and eliminating spoofing earlier and faster.

## 1. Introduction

Global satellite navigation systems (GNSSs) are widely used in civil aviation, transportation, power, finance and other fields [1]. However, due to the low power of their navigation signals, they are easily affected by various interferences, such as spoofing and suppression. Spoofing interference is a great threat to GNSS security [2,3]. In 2001, a report on the vulnerability of GNSS was assessed by the US Department of transportation, which pointed out that there are serious security risks in transportation systems [4]. In 2009, the US naval surface operations center issued a report on the problem of GNSS spoofing interference [5], stating that GNSS should not only focus on positioning accuracy, but also on application security [6].

Signal processing algorithms have been studied as a means to detect spoofing interference. The difference in a Doppler shift between a satellite’s navigation signal and spoofing to identify the spoofing [7,8]. Monitoring a received signal’s power, carrier noise ratio and noise level can also be used to detect spoofing [9,10,11]. A new signal-quality assessment model has been proposed to detect and identify spoofing [12] that can work well even when the strength of a received signal’s spoofing and authentic signal are very close to each other. However, the performance of this algorithm may deteriorate when the code phase differences between authentic signals and spoofing signals are <1.5 chips and the Doppler frequency differences between authentic signals and spoofing signals are relatively small. Sensitivity models have been formulated that include a vestigial signal-to-interference and noise ratio (SINR) that is quantified and characterized to detect the probability of a false alarm [13]. Power-distortion detectors identify spoofing by analyzing the distortion of the received power via a correlation function [14]. This enables civil global positioning system receivers and other civil global navigation satellite system receivers to reliably detect carry-off spoofing and jamming.

Data processing algorithms have also been studied as a means of detecting spoofing. When an antenna is rotating, the power measurements of the spoofing signals coming from the same direction change similarly and the correlation coefficients between them are close to 1, but the power measurements of the authentic signals are uncorrelated [15]. A new approach for GPS spoofing detection based on a multi-layer neural network (NN) whose inputs are indices of features is presented in [16]. This method demonstrated adequate detection accuracy from the NN with a short detection time. The autonomous integrity information of the receiver can also be used to detect and identify spoofing [17], and it is able to resist attacks from one or many spoofing satellites. Inertial Navigation System (INS)/GNSS integrated navigation can also be used to detect and identify spoofing, using a Kalman filter (KF) and non-linear approximation techniques such as an extended Kalman filter (EKF), (Sigma Point Kalman Filter (SPKF)) or divided difference filters (DDF) [18,19].

Finally, suppression algorithms using multiple antennas have been studied as a means of detecting spoofing interference. If a spoofing transmitter transmits spoofing signals from several different satellites at the same time, the spatial correlation of each spoofing signal can be detected by multi antenna direction finding (similar to an interferometer) [20,21]. Since the baseline between the antennas is known, the direction of arrival of the received signal can be obtained using the same satellite to reach the antenna carrier phase difference [22]; if different satellite signals come from the same direction, it can be judged that their own party has been deceived. After determining the direction of the spoofing, the spoofing can be eliminated or suppressed. A new approach [23] has also been proposed combing a generalized side-lobe canceller (GSC) and space-time adaptive faltering (which is used to suppress spoofing). In addition, an encryption algorithm based on a signal’s authentication sequence has been analyzed that can resist a spoofing signal’s attack [24,25], but the signal format needs to be modified, as it is not suitable for the GNSS civil navigation signal that has been applied at present.

In the following sections, an adaptive spoofing-interference suppression algorithm is proposed for satellite navigation based on a multi antenna array. The cross-correlation gain of the spread spectrum signal received by the array is determined to be higher than the noise level, and the direction of the received signal does not need to be estimated, as this algorithm can realize the adaptive filtering of satellite navigation spoofing. It reduces the complexity of satellite navigation spoofing suppression and improves its performance. The main contributions of this paper are as follows:

1. An adaptive spoofing interference suppression algorithm is proposed for satellite navigation based on a multi antenna array. This algorithm improves the real-time performance of spoofing suppression and reduces the complexity of spoofing interference suppression.
2. A cross-correlation model of satellite navigation spoofing signals received by multiple elements is established. The feasibility of using the spread spectrum signals received between different array elements as a cross-correlation by which to suppress spoofing interference is analyzed.
3. The complexity of the implementation of the adaptive spoofing suppressor for satellite navigation based on a multi antenna array is analyzed, and the performances of the two algorithms are verified by comparing them with the algorithm that first finds the direction of a signal and then suppresses it. The location performances of the two algorithms are tested under static and dynamic conditions.

## 2. Spoofing Suppression Algorithm

### 2.1. Signal Receiving Model of a Multiple Antenna Array for Spoofing

Assume an arbitrary M-element antenna array configuration. In this configuration, one antenna is chosen as the reference antenna. Without loss of generality, assume that the navigation satellite signals can be received at a certain time L. The received signal on the m array element can now be expressed as

(1) $$x_{m}\left(t\right)=\sum_{i=1}^{L}sa_{i}\left(t\right)a_{m}(\theta_{i})+n\left(t\right)$$

where n(t) is additive white Gaussian noise, $a_{m}\left(\theta_{i}\right)$ is the direction vector of satellite i, $\theta_{i}$ is the direction of arrival of the i th satellite signal, $\tau_{i}$ is the delay of the ith satellite signal $sa_{i}\left(t\right)$ is the navigation signal of the ith satellite received, and t is the signal arrival time. The navigation signal composition is as follows:

(2) $$sa_{i}\left(t\right)=\sqrt{P_{i}}b_{i}\left(t-\tau_{i}\right)c_{i}\left(t-\tau_{i}\right)$$

where $P_{i}$ is the total transmit power of the ith satellite, $b_{i}\left(t\right)\in [0,T_{b}]$ is the ith satellite data bit, and $c_{i}$ is the binary spreading (ranging) code of the ith satellite [26].

If the received signal contains a spoofing signal, the received signal can be written as

(3) $$x_{m}\left(t\right)=\sum_{i=1}^{L}sa_{i}\left(t\right)a_{m}(\theta_{i})+\sum_{k=1}^{K}sp_{k}(t)a_{m}(\theta_{k})+n\left(t\right)$$

where $\theta_{k}$ is the direction of the spoofing signal, K is the number of spoofings, $sp_{k}\left(t\right)$ is the spoofing signal for the kth satellite, $sp_{k}\left(t\right)=\sqrt{P_{sk}}b_{k}\left(t-\tau_{sk}\right)c_{k}\left(t-\tau_{sk}\right)a_{m}(\theta_{k})$, $\tau_{sk}$ is the delay of the spoofing signal, and $c_{k}$ is the binary spreading (ranging) code of the kth spoofing signal. Spoof signals are often similar to currently visible satellite signals. The received signal of the mth array element can now be written as

(4) $$x_{m}\left(t\right)=\sum_{i=1}^{N}\sqrt{P_{i}}b_{i}\left(t-\tau_{i}\right)c_{i}\left(t-\tau_{i}\right)a_{m}(\theta_{i})+\sum_{k=1}^{K}sp_{k}(t)+n\left(t\right)$$

M receives spatial samples of authentic and spoofing signals impinging on the antenna array before despreading, which can be written in a matrix form as

(5) $$X_{M}=\left[\begin{array}{c} x_{1}\left(t\right)\\ x_{2}\left(t\right)\\ \vdots \\ x_{M}\left(t\right) \end{array}\right]=\left[\begin{array}{c} \sum_{i=1}^{L}sa_{i}\left(t\right)a_{1}(\theta_{i})\\ \sum_{i=1}^{L}sa_{i}\left(t\right)a_{2}(\theta_{i})\\ \vdots \\ \sum_{i=1}^{L}sa_{i}\left(t\right)a_{M}(\theta_{i}) \end{array}\right]+\left[\begin{array}{c} \sum_{i=1}^{L}sp_{i}\left(t\right)a_{1}(\theta_{i})\\ \sum_{i=1}^{L}sp_{i}\left(t\right)a_{2}(\theta_{i})\\ \vdots \\ \sum_{i=1}^{L}sp_{i}\left(t\right)a_{M}(\theta_{i}) \end{array}\right]+\left[\begin{array}{c} n_{1}\left(t\right)\\ n_{2}\left(t\right)\\ \vdots \\ n_{m}\left(t\right) \end{array}\right]$$

The use of arrays to receive satellite navigation signals can enhance or suppress signals from different directions, thereby achieving suppression of satellite navigation interference signals.

### 2.2. Suppressing Spoofing Based on Direction of Arrival (DOA) 

Filtering (despread) the received signal x(t) of the array according to the spreading code $c_{i}\left(t-\tau_{i}\right)$ of the ith desired satellite results in the nth bit of the processed signal being written as follows:

(6) $$y_{mi}\left(n\right)=\frac{1}{\sqrt{T_{b}}}\int_{(n-1)T_{b}+\tau_{1}}^{nT_{b}+\tau_{1}}x\left(t\right)c_{i}\left(t-\tau_{i}\right)dt=\sqrt{T_{b}P_{i}}b_{i}\left(n\right)e^{-j\varphi (\theta )}a_{m}\left(\theta_{i}\right)+\sqrt{T_{b}P_{si}}b_{i}\left(n\right)e^{-j\varphi (\beta_{})}a_{m}\left(\beta_{i}\right)+n\left(t\right)$$

where $T_{b}$ is the binary spreading (ranging) code period, $c_{i}$ is the binary spreading (ranging) code of the ith spoofing signal, $P_{i}$ is the navigation signal gain, $P_{si}$ is the gain of spoofing, $b_{i}\left(n\right)$ is the ith satellite data bit, $\theta_{i}$ is the direction of arrival of the ith navigation signal, and $\beta_{i}$ is the direction of arrival of the ith spoofing signal.

Let $y_{i}\left(n\right)={\left[y_{1i},y_{2i},\cdots ,y_{Mi}\right]}^{H}$, $A\left(\theta_{i}\right)={\left[a_{1}\left(\theta_{i}\right),a_{2}\left(\theta_{i}\right),\cdots ,a_{M}\left(\theta_{i}\right)\right]}^{H}$. Then the covariance matrix of the despread signal $y_{i}$ can be approximated as

(7) $$R_{y_{i}y_{i}}=\frac{1}{T_{c}}E\left\{y_{i}\left(n\right)y_{i}^{*}\left(n\right)\right\}=GP_{i}A(\theta_{i})A^{H}(\theta_{i})+GP_{si}A(\beta_{i})A^{H}(\beta_{i})+\sigma_{n}^{2}I$$

where $\sigma_{n}^{2}$ is the variance of thermal noise, I is the unit matrix, $T_{c}$ is the chip interval, and $G=\frac{T_{b}}{T_{c}}$ is the spreading gain.

After despreading, the signal power becomes G times that of the original. With reference to the general satellite transmitted signal, the gain G of the despreading processing is determined to be about 43 db. Generally, the level of the satellite navigation signal reaching the antenna interface is about −20 dB, and spoofing is usually about 5–10 dB higher than the satellite signal in order to achieve a good spoofing effect. After dispreading, the signal power is 23–30 dB higher than the noise power. Therefore, the traditional Direction of Arrival (DOA) estimation algorithm [27,28,29] can be used to measure the direction of arrival of the spoofing. In this paper, we use the multiple signal classification (MUSIC) algorithm [30] as an example to introduce the process of estimating the direction of arrival of spoofing.

Assume that there is one desired signal and K spoofing. After performing the feature decomposition on the covariance matrix (7), $R_{y_{i}y_{i}}$ can be expressed as

(8) $$R_{y_{i}y_{i}}=\sum_{i=1}^{K+1}\lambda_{i}u_{i}u_{i}^{H}+\sigma_{n}^{2}\sum_{i=K+2}^{M}u_{i}u_{i}^{H}$$

where eigenvalues of the covariance matrix $\lambda_{1}\geq \lambda_{2}\geq \cdots \lambda_{K+1}>\lambda_{K+2}=\cdots =\lambda_{M}=\sigma_{n}^{2}$ are the corresponding M eigenvalues, of which the corresponding feature vector is $u_{i}$ (i=1,2,⋯,M). This is written as

(9) $$D_{s}=diag\left(\lambda_{1},\lambda_{2},\cdots ,\lambda_{K+1}\right)$$

(10) $$D_{n}=diag\left(\lambda_{K+2},\lambda_{K+3},\cdots ,\lambda_{M}\right)$$

The corresponding signal subspace of the large eigenvalue expansion is $U_{s}=\left[u_{1},u_{2},\cdots ,u_{K+1}\right]$, and the noise subspace is $U_{N}=\left[u_{K+2},u_{K+3},\cdots ,u_{M}\right]$. The signal subspace and noise subspace are orthogonal to each other.

Simultaneously, the array direction vector of the received signals is also orthogonal to the noise subspace. As such, the spatial spectrum function of the MUSIC algorithm $U_{N}^{H}A\left(\theta_{i}\right)=0$ can be expressed as:

(11) $$P_{MUSIC}\left(\theta \right)=\frac{1}{A^{H}\left(\theta \right)U_{N}U_{N}^{H}A\left(\theta \right)}$$

Estimating the direction of arrival of the spoofing signal and the navigation signal is achieved via Equation (11).

After determining the direction of arrival of the received signals, spoofing and navigation signals are identified based on their energy and direction angle. The array flow pattern $B_{S}=\left[\begin{array}{cccc} a({\overset{⌢}{\theta }}_{1}^{s}) & a({\overset{⌢}{\theta }}_{2}^{s}) & \cdots & a\left(\theta_{M}^{s}\right) \end{array}\right]$ can be obtained according to the array steering vector of the spoof signal. The spoofing signal subspace [31] can now be expressed as

(12) $$U_{S_{0}}=B_{S}{\left(B_{S}^{H}B_{S}\right)}^{-1}B_{S}$$

According to the orthogonality of the signal subspace and the noise subspace, the noise subspace can be obtained:

(13) $$U_{N_{0}}=I-U_{S_{0}}=I-B_{S}{\left(B_{S}^{H}B_{S}\right)}^{-1}B_{S}$$

The optimization problem of beam weighting minimizes the power of residual interference and noise in the output. Since the optimal weight vector does not change the power of the target signal, the output signal-to-interference and noise ratio can be maximized. Using the Lagrangian multiplier algorithm, the solution of the optimal filter processor can be expressed as

(14) $$w_{\mathrm{opt}}^{}={\left[s^{H}U_{N_{0}}s\right]}^{-1}U_{N_{0}}s$$

where s is the constraint vector of M×1; without constraint, the vector is $s={\left[1,0,\cdots ,0\right]}^{H}$. When the direction of arrival of the satellite signal is known, the value of constraint vector can be determined in order to enhance the satellite signal using $s={\left[1,a_{1}\left(\alpha \right),\cdots ,a_{M}\left(\alpha \right)\right]}^{H}$, where α is the satellite navigation signal constraint direction. The output of filtering processing is

(15) $$y_{\mathrm{out}}=w_{\mathrm{opt}}^{H}X_{M}$$

where y is the signal after spoofing suppression.

**Figure 1:**
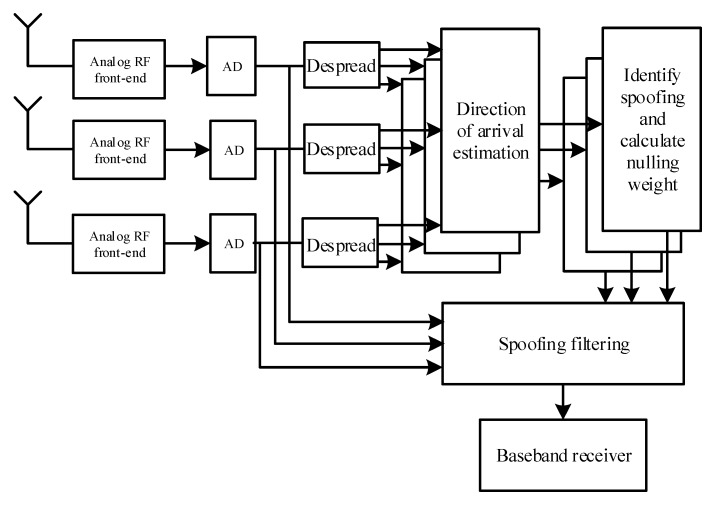
Suppressing spoofing based on Direction of Arrival (DOA).

A block diagram of the implementation of the spoofing suppression algorithm based on DOA is shown in Figure 1.

The implementation process of this algorithm is complicated and not conducive to real-time implementation. The implementation process of the algorithm can be described as follows:
** Algorithms:**The received signal of the array antenna is $X_{M}=\left[x_{1}\left(t\right),x_{2}\left(t\right),\cdots x_{M}\left(t\right)\right]$Despreading the signals received by multiple antennas for one satellite is expressed as $y_{mi}\left(n\right)=\frac{1}{\sqrt{T_{b}}}\int_{(n-1)T_{b}+\tau_{1}}^{nT_{b}+\tau_{1}}x\left(t\right)c_{i}\left(t-\tau_{i}\right)dt=\sqrt{T_{b}P_{i}}b_{i}\left(n\right)e^{-j\varphi (\theta )}a_{m}\left(\theta_{i}\right)+\sqrt{T_{b}P_{si}}b_{i}\left(n\right)e^{-j\varphi (\beta_{})}a_{m}\left(\beta_{i}\right)+n\left(t\right)$The autocorrelation matrix $R_{y_{i}y_{i}}$ of the despreading signal is determined along with the eigenvalues of the matrix. The signal subspace $U_{s}=\left[u_{1},u_{2},\cdots ,u_{K+1}\right]$ and noise subspace $U_{N}=\left[u_{K+2},u_{K+3},\cdots ,u_{M}\right]$ are built.The spatial spectrum function $P_{MUSIC}\left(\theta \right)=\frac{1}{A^{H}\left(\theta \right)U_{N}U_{N}^{H}A\left(\theta \right)}$ is constructed. The spectrum peak is determined, and discrimination of the spoofing and navigation signals is carried out based on search spectrum peak-to-peak size, number and direction of incidence angle.Subspace $U_{S_{0}}=B_{S}{\left(B_{S}^{H}B_{S}\right)}^{-1}B_{S}$ of the spoofing signal is constructed based on array flow pattern $B_{S}=\left[\begin{array}{cccc} a({\overset{⌢}{\theta }}_{1}^{s}) & a({\overset{⌢}{\theta }}_{2}^{s}) & \cdots & a\left(\theta_{M}^{s}\right) \end{array}\right]$ of the spoofing signal incidence angle. The corresponding noise subspace $U_{N_{0}}=I-U_{S_{0}}=I-B_{S}{\left(B_{S}^{H}B_{S}\right)}^{-1}B_{S}$ is determined, and the optimal weight of spoofing suppression without constraints or satellite signal direction constraints is identified and shown as $w_{\mathrm{opt}}^{}={\left[s^{H}U_{N_{0}}s\right]}^{-1}U_{N_{0}}s$.According to the optimal weight vector, the received signal of array antenna is filtered, and the filtered navigation signal is obtained, shown as $y_{\mathrm{out}}=w_{\mathrm{opt}}^{H}X_{M}$. The filtered signal $y_{out}$ is sent to the baseband receiver for acquisition tracking.

### 2.3. Adaptive Spoofing Suppression Algorithm (Assa)

Without estimating the direction of arrival of the spoofing signal, adaptive spoofing suppression is an effective way to reduce the complexity of the spoofing interference suppression.

According to Equation (1), the cross-correlation of received signals from different array elements can be defined as:

(16) $$q_{m}\left(n\right)=\frac{1}{\sqrt{T_{b}}}\int_{(n-1)T_{b}+\tau_{1}}^{nT_{b}+\tau_{1}}x_{m}\left(t\right)x_{q}\left(t\right)dt$$

where $x_{q}\left(t\right)$ is the received signal of the reference array element, and $x_{m}\left(t\right)$ is the received signal of the mth array element. Expanding on this, Equation (16) can be written as:

(17) $$\begin{array}{c} q_{m}\left(n\right)=\frac{1}{\sqrt{T_{b}}}\int_{(n-1)T_{b}+\tau_{1}}^{nT_{b}+\tau_{1}}\left(\sum_{i=1}^{L}sa_{i}\left(t\right)a_{m}(\theta_{i})+\sum_{k=1}^{K}sp_{k}\left(t\right)a_{m}(\theta_{k})+n\left(t\right)\right)\left(\sum_{i=1}^{L}sa_{i}\left(t\right)a_{q}(\theta_{i})+\sum_{k=1}^{K}sp_{k}\left(t\right)a_{q}(\theta_{k})+n\left(t\right)\right)dt\\ \approx \frac{1}{\sqrt{T_{b}}}\int_{(n-1)T_{b}+\tau_{1}}^{nT_{b}+\tau_{1}}\left(\sum_{i=1}^{L}sa_{i}\left(t\right)a_{m}(\theta_{i})+\sum_{k=1}^{K}sp_{k}\left(t\right)a_{m}(\theta_{k})\right)\left(\sum_{i=1}^{L}sa_{i}\left(t\right)a_{q}(\theta_{i})+\sum_{k=1}^{K}sp_{k}\left(t\right)a_{q}(\theta_{k})\right)dt+n\left(t\right) \end{array}$$

The low cross-correlation gain between different satellite signals is due to general pseudo-code characteristics, and the spoofing signal is less than the number of currently visible navigation satellites, K≤L. Equation (17) can therefore be written as:

(18) $$\begin{array}{cc} q_{m}\left(n\right)= & \frac{1}{\sqrt{T_{b}}}\int_{(n-1)T_{b}+\tau_{1}}^{nT_{b}+\tau_{1}}\left(\sum_{i=1}^{L}sa_{i}^{2}\left(t\right)a_{m}(\theta_{i})a_{q}(\theta_{i})\right)dt+\frac{1}{\sqrt{T_{b}}}\int_{(n-1)T_{b}+\tau_{1}}^{nT_{b}+\tau_{1}}\left(\sum_{k=1}^{K}sp_{k}^{2}\left(t\right)a_{m}(\theta_{k})a_{q}(\theta_{k})\right)dt+\cdots \\ & \frac{1}{\sqrt{T_{b}}}\left(\int_{(n-1)T_{b}+\tau_{1}}^{nT_{b}+\tau_{1}}\left(\sum_{i=1}^{L}sa_{i}^{}\left(t\right)a_{m}(\theta_{i})\right)\left(\sum_{k=1}^{K}sp_{k}^{}\left(t\right)a_{q}(\theta_{k})\right)+\left(\sum_{i=1}^{L}sa_{i}^{}\left(t\right)a_{q}(\theta_{i})\right)\left(\sum_{k=1}^{K}sp_{k}^{}\left(t\right)a_{m}(\theta_{k})\right)\right)dt+n\left(t\right) \end{array}$$

Because real satellite signals come from different directions, it can be assumed that all spoofing signals come from the same source, such as $\theta_{k}=\theta_{K}(k=1,2,\cdots K)$. t can be further be simplified as:

(19) $$\begin{array}{l} q_{m}\left(n\right)=\frac{1}{\sqrt{T_{b}}}\int_{(n-1)T_{b}+\tau_{1}}^{nT_{b}+\tau_{1}}\left(\sum_{i=1}^{L}sa_{i}^{2}\left(t\right)a_{m}(\theta_{i})a_{q}(\theta_{i})\right)dt+\frac{a_{m}(\theta_{K})a_{q}(\theta_{K})}{\sqrt{T_{b}}}\int_{(n-1)T_{b}+\tau_{1}}^{nT_{b}+\tau_{1}}\left(\sum_{k=1}^{K}sp_{k}^{2}\left(t\right)\right)dt+\cdots \\ \frac{1}{\sqrt{T_{b}}}\left(\int_{(n-1)T_{b}+\tau_{1}}^{nT_{b}+\tau_{1}}a_{q}(\theta_{k})\left(\sum_{i=1}^{K}sa_{i}^{}\left(t\right)sp_{i}^{}\left(t\right)a_{m}(\theta_{i})\right)+a_{m}(\theta_{K})\left(\sum_{i=1}^{K}sa_{i}^{}\left(t\right)sp_{i}^{}\left(t\right)a_{q}(\theta_{i})\right)\right)dt+n\left(t\right) \end{array}$$

Generally, the received power of the spoofing is about 5–10 dB higher than that of the satellite navigation signal. In other words, the spoofing gain is about 10–20 dB higher than the navigation signal after correlation despreading. The power of the satellite navigation signal to the receiving antenna is usually about 20 dB less than the noise. Taking the BeiDou B1 signal as an example, the gain of correlation despreading is about 21 dB. Different satellite navigation signals arrive from different directions, and array signal processing will restrict signal enhancement from a certain direction; therefore, the signals energy will not exceed the noise after cross-correlation between different channels. The cross-correlation gain of the spoofing signal is 10–20 dB higher than that of the navigation signal, which is to say that the level of the cross-correlation spoofing signal is above the noise. If the spoofing signal is a single-address broadcast spoofing signal, the array can receive multiple spoofing signals to achieve a superposition from the same direction.

Therefore, formula (19) can be approximately equal to:

(20) $$q_{m}\left(n\right)\approx \frac{a_{m}(\theta_{K})a_{q}(\theta_{K})}{\sqrt{T_{b}}}\int_{(n-1)T_{b}+\tau_{1}}^{nT_{b}+\tau_{1}}\left(\sum_{k=1}^{K}sp_{k}^{2}\left(t\right)\right)dt+n\left(t\right)$$

By expanding the components of Equation (20), we can get:

(21) $$q_{m}\left(n\right)=\sqrt{T_{b}}a_{m}(\theta_{K})a_{q}(\theta_{K})\sum_{k=1}^{K}P_{sk}b_{k}\left(n\right)+n\left(t\right)$$

**Figure 2:**
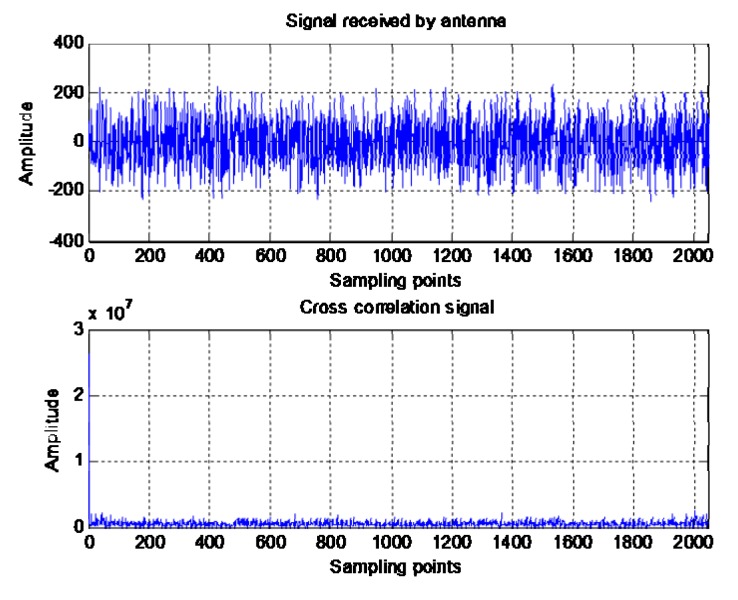
Signal comparison before and after cross-correlation.

The cross-correlation gain generated by cross-correlation processing between the signals received by different antenna elements is shown in the Figure 2:

The signals received by the array elements are completely submerged under the noise, and the adaptive notch algorithm cannot be used to achieve the suppression of spoofing. When the signals received by the two array elements are cross-correlated and coherently accumulated, a peak can be formed that is equivalent to that the energy of the received signal and higher than the noise. Therefore, the adaptive notch algorithm can be used to suppress spoofing.

Assume the cross-correlation data length is N; the received signal after cross-correlation can be expressed as $Q_{N}=\left[q_{1}\;q_{2}\cdots q_{M}\right]$. The covariance matrix after cross-correlation can now be expressed as

(22) $$R_{Q}=(\sum_{n=1}^{N}Q_{N}*Q_{N}{}^{H})/N$$

After obtaining the covariance matrix after cross-correlation, the optimal weight of the adaptive notch can be calculated according to the following formula:

(23) $$w_{\mathrm{opt}}=R_{Q}^{-1}*s$$

Without directional constraints, $s={\left[1,0,\cdots ,0\right]}^{H}$. In practical applications, satellite signals usually come from the area with a high elevation angle, while spoofing usually occurs in a place with a low elevation due to the limitations of the actual environment angle. Constraints are put on specific directions, and the value of the constraint vector is determined as $s={\left[1,a_{1}\left(\alpha \right),\cdots ,a_{M}\left(\alpha \right)\right]}^{H}$, where α is the signal-constraint enhancement direction.

The output of the adaptive filtering processing is

(24) $$y_{\mathrm{out}}=w_{\mathrm{opt}}^{H}X_{M}$$

**Figure 3:**
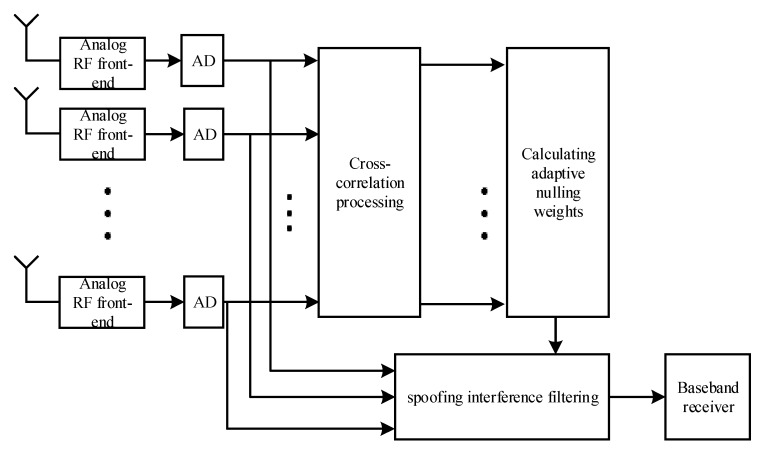
Adaptive spoofing suppression.

Figure 3 shows the processing flow of the adaptive spoofing suppression algorithm, which does not require a direction to be established. Multiple spoofing interferences can be suppressed by the adaptive nulling algorithm after a cross-correlation. The algorithm flow can be described as follows:
** Algorithms:**The array antenna receiving signal is $X_{M}=\left[x_{1}\left(t\right),x_{2}\left(t\right),\cdots x_{M}\left(t\right)\right]$.Any one of these can be selected as a cross-correlation reference signal using $x_{q}\left(t\right)=x_{1}\left(t\right)$.The cross-correlation vector of each signal and the reference signal are calculated for m=1,2,⋯,M, $q_{m}=\frac{1}{\sqrt{T_{b}}}\int_{(n-1)T_{b}+\tau_{1}}^{nT_{b}+\tau_{1}}x_{m}\left(t\right)x_{q}\left(t\right)dt$, end.The cross-correlation matrix $Q_{N}=\left[q_{1}\;q_{2}\cdots q_{M}\right]$ is calculated. The sample lengths of each channel in the N-point covariance matrix are $R_{Q}=(\sum_{n=1}^{N}Q_{N}*Q_{N}{}^{H})/N$.The constraint vector of the adaptive notch is generated according to constraint conditions s.The weight vector of adaptive notch is calculated by $w_{\mathrm{opt}}=R_{Q}^{-1}*s$.The array’s received signals are filtered by the calculated weights: $y_{\mathrm{out}}=w_{\mathrm{opt}}^{H}X_{M}$.

### 2.4. Complexity Analysis

There are three steps in the computation process of the spoofing suppression algorithm based on DOA. The first is despreading the received signal, assuming that the signal is despread in the form of Fast Fourier Transformation (FFT) transform (the computation amount of one channel is $\frac{3N}{2}\log_{2}N+N$, the operation amount of M channels is $M\left(\frac{3N}{2}\log_{2}N+N\right)$, N is the number of sampling points). The second step is estimating the direction of arrival after despreading, assuming the MUSIC algorithm is adopted (the computation amount is $M^{3}+NM^{2}+(2M-1)MP$, P is the search times of the algorithm in azimuth and pitch direction). The third step is to establish fixed-direction nulling (the operation amount of this part is $M^{3}+6M^{2}+5M+1$). The total computation amount is $Q_{1}=M\left(\frac{3N}{2}\log_{2}N+N\right)+2M^{3}+6M^{2}+5M+1+NM^{2}+\left(2M-1\right)MP$. Tf K spoofing signals are suppressed, the amount of computation needs to be increased K times.

The calculation process of the adaptive spoofing suppression algorithm based on a multiple antenna array proposed in this paper is divided into two parts: The first is the amount of cross-correlation calculations (the computation amount of M channels is $M\left(\frac{3N}{2}\log_{2}N+N\right)$). The second is adaptive nulling generation (the amount of computation is $M^{3}+M^{2}$) The calculation amount of the algorithm in this paper is $Q_{2}=M\left(\frac{3N}{2}\log_{2}N+N\right)+M^{3}+M^{2}$. An increase in the amount of spoofing will not cause an increase in the amount of computation.

Comparing the computation of the two algorithms, $Q_{2}<<Q_{1}<KQ_{1}$, and as the number of array elements increases the gap between the two algorithms increases geometrically. The spoofing suppression algorithm based on DOA needs to capture and track the signal first, which is equivalent to a baseband navigation receiver behind each channel, and the synchronization of time and code between different receivers is complex. The ASSA based on a multiple antenna array proposed in this paper is implemented before acquisition and tracking, and has good real-time performance. The back end only needs a baseband navigation receiver, and the amount of equipment is small. Therefore, the algorithm proposed in this paper can greatly reduce the computation of array deception suppression.

## 3. Implementations and Evaluation

**Figure 4:**
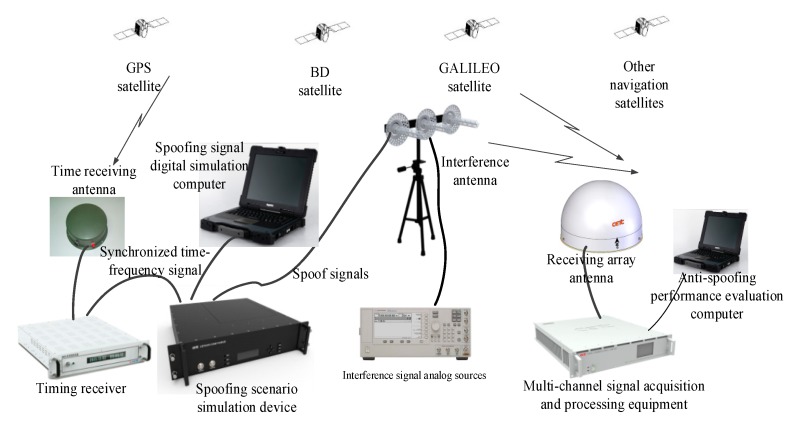
Composition of the wireless anti-spoofing test.

In order to verify the spoofing suppression performance of the algorithm proposed in this paper, we needed to build a satellite navigation anti-spoofing verification environment and collect spoofing data for verification. The equipment components of the verification environment included a spoofing scenario simulation device, spoofing signal digital simulation computer, interference signal analog sources, receiving array antenna, multiple channel signal acquisition and processing equipment, anti-spoofing performance evaluation computer, timing receiver and interference transmitting antenna, and resource allocation within the system needed to be optimized [32]. The test connection relationship is shown in Figure 4, which includes purchased commercial equipment and self-developed equipment.

**Figure 5:**
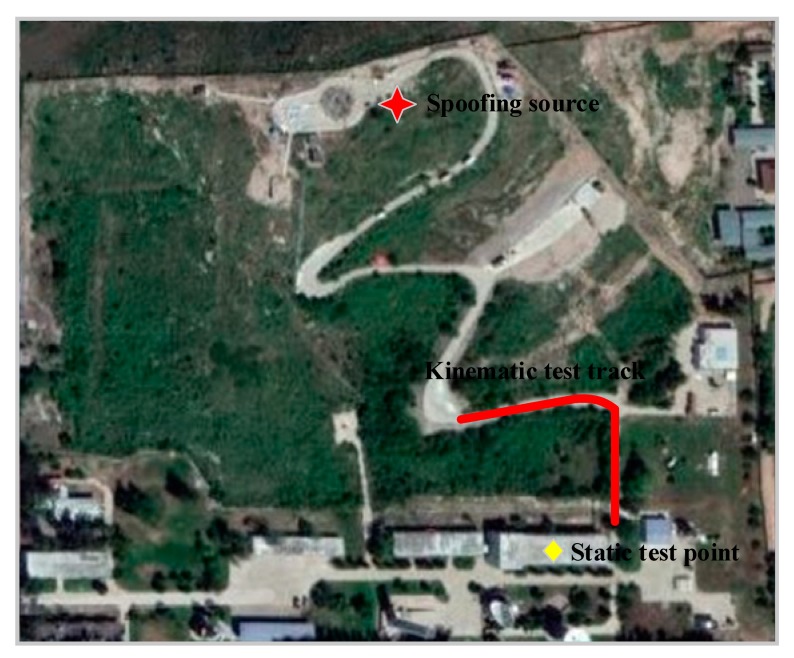
Wireless anti-spoofing test environment.

An anti-spoofing test environment was set up at an outdoor test site. The test site was on a hill more than 100 m high, and about 30 m above the flat ground below the hill. The spoofing interference source was installed at the top of the mountain, and could simultaneously transmit eight spoofing interference signals. The spoofing signal had the same pseudo-code structure as the satellite signal visible in the current area. The test trajectory is shown by the red curve in Figure 5. The receiving array used a seven-element Y-shaped circular array, and used signal acquisition and processing equipment to collect satellite navigation and spoofing signals. The test schematic is shown in Figure 5, with a background taken from Google Maps.

The spoofing simulator generated a spoofing signal with the same pseudo code as the visible satellite in the current area. On the basis of synchronization with the navigation time of the satellite in the sky, eight spoofing signals were transmitted simultaneously by means of single-point broadcasting. The power at the receiving end of the spoofing signal was 5–15dB higher than that of navigation signal.

This test site was located in Cuiwei hill, Luquan District, Shijiazhuang, Hebei Province. The spoofing interference suppression test was completed in October 2019. The weather on the test day was clear, and the ionosphere and troposphere conditions were relatively stable.

### 3.1. Performance Analysis

The BeiDou B1 signal was used as an example to analyze the spoofing interference suppression performance of the spoofing suppression algorithm. The test equipment composition and test environment are shown in Figure 4 and Figure 5. The signal acquisition and processing equipment saved the collected signals into files, and Matlab was used to analyze the spoofing suppression performance of the two algorithms.

**Figure 6:**
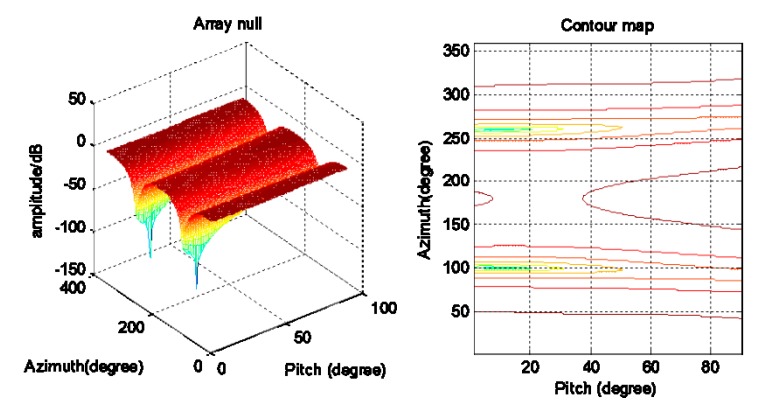
Spoofing suppression based on DOA.

**Figure 7:**
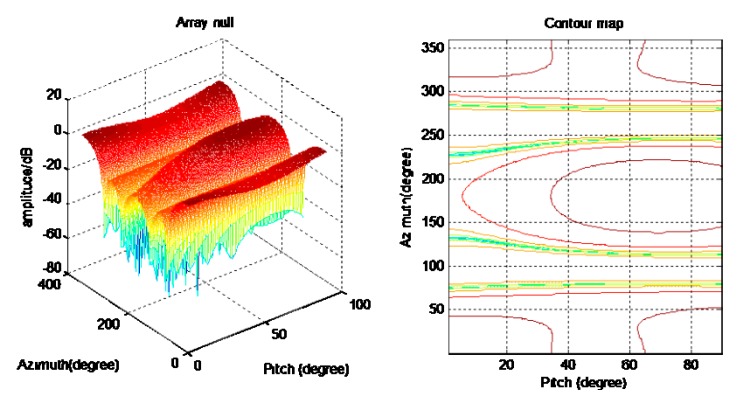
Adaptive spoofing suppression.

**Figure 8:**
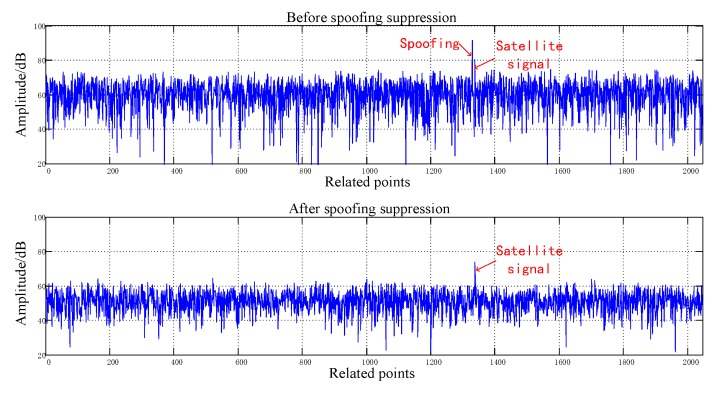
Comparison before and after spoofing suppression (1).

Figure 6 and Figure 7 are a spatial spectrum diagram and a gradient projection diagram of the spoofing suppression algorithm based on DOA, respectively. Figure 8 shows that the algorithm only produces nulls in the direction of spoofing, while the other directions are relatively flat. There is also less impact on the reception of navigation signals as the spoofing suppression is completed. This algorithm has the same implementation process for the suppression of spoofing in different satellites, but to save space the processing of other satellite channels is not listed one by one. This algorithm requires a large amount of calculations and a complicated implementation, but the advantage is that the algorithm can achieve spoof interference suppression in which the direction of the spoof interference emission is greater than the number of array elements because the satellite channels are processed separately and not limited by the number of array elements.

**Figure 9:**
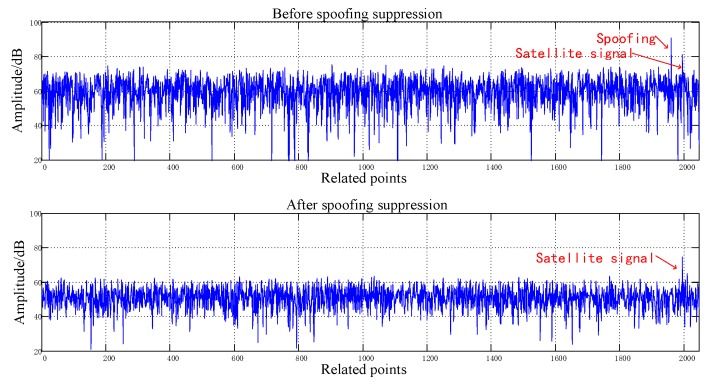
Comparison before and after spoofing suppression (2).

Figure 7 is the spatial spectrum and gradient projection of the ASSA. Figure 9 shows that the algorithm can also form nulls in the direction of spoofing, but that compared with the algorithm that finds the direction first then suppresses backwards, the formed nulls are shallower. The zero trap formed by this algorithm is flat, and there are many redundant nulls, but the algorithm can also achieve a suppression of spoofing.

Figure 8, Figure 9, Figure 10, Figure 11, Figure 12, Figure 13, Figure 14 and Figure 15 correspond respectively to the received signals of the eight satellites being cheated, and show comparisons of the correlated despread signal before and after the spoofing suppression.

**Figure 10:**
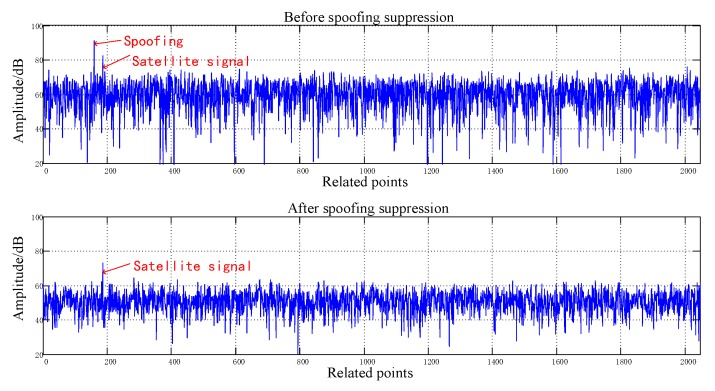
Comparison before and after spoofing suppression (3).

**Figure 11:**
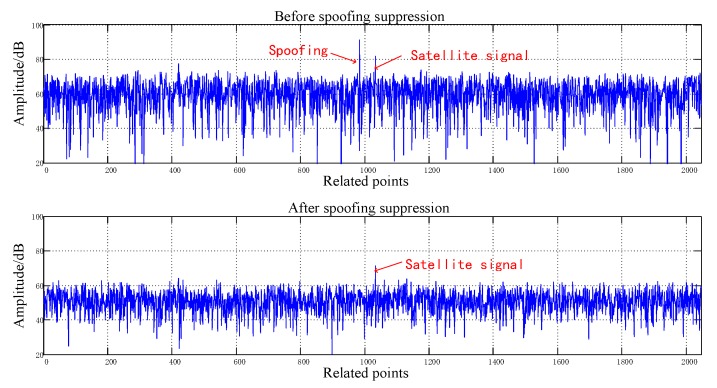
Comparison before and after spoofing suppression (4).

**Figure 12:**
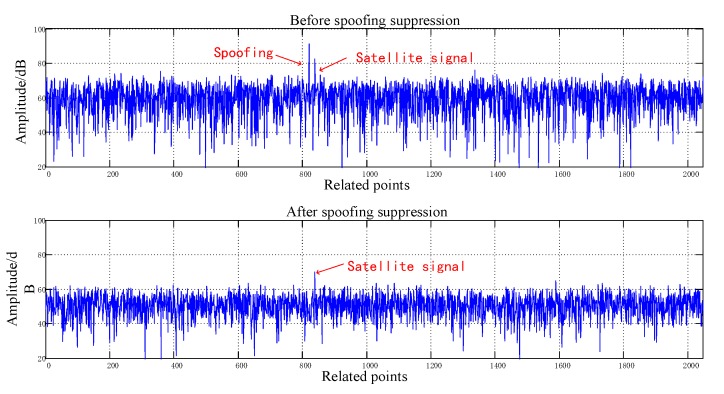
Comparison before and after spoofing suppression (5).

**Figure 13:**
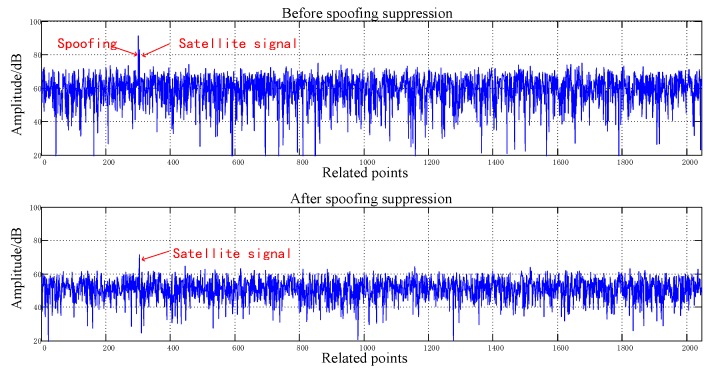
Comparison before and after spoofing suppression (6).

**Figure 14:**
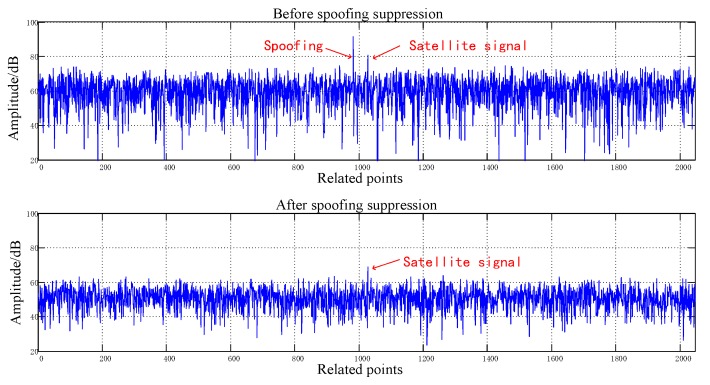
Comparison before and after spoofing suppression (7).

**Figure 15:**
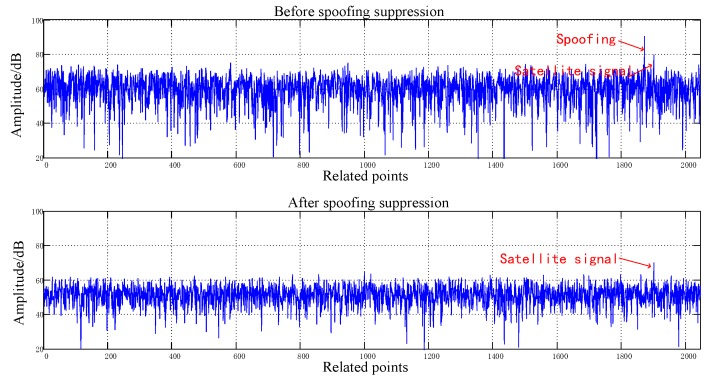
Comparison before and after spoofing suppression (8).

**Figure 16:**
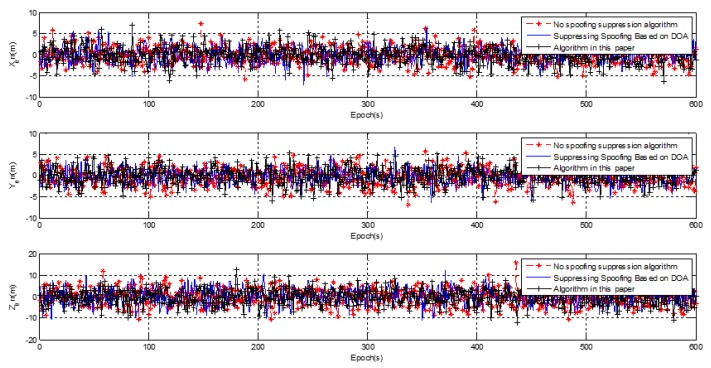
Static positioning error without spoofing.

**Figure 17:**
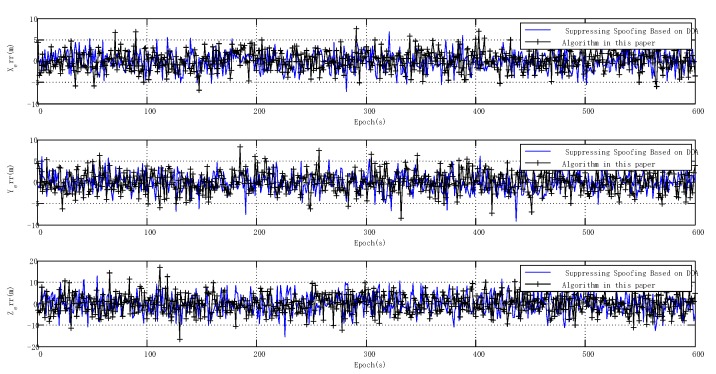
Static positioning error with spoofing.

The ASSA can achieve the simultaneous suppression of multiple spoofing interferences. Figure 10, Figure 11, Figure 12, Figure 13, Figure 14, Figure 15, Figure 16 and Figure 17 show that there were two signals before suppression, a spoofing signal and navigation signals, and that only one navigation signal remained after suppression. Although the algorithm processing process causes the loss of satellite signal energy, the carrier-to-noise ratio is reduced by less than 1 dB, which has little effect on the receiver’s acquisition and tracking.

### 3.2. Static Test Results

To validate the usability of the ASSA on the positioning performance, the static navigation performance of the general navigation software receiver was compared with the software receiver loaded with the spoofing suppression algorithm based on DOA and the software receiver loaded with the ASSA. In the experiment, the receiving antenna was placed at a known point calibrated in advance, and the coordinates of the local Cartesian coordinate system were (4235045.81 m, 530526.85 m, 97.84 m). Figure 16 shows the static positioning error without spoofing interference.

Without spoofing interference, the navigation signal had little effect on the performance of navigation and positioning after being processed by the two algorithms introduced in this paper. In order to more intuitively evaluate the impact of the two algorithms on positioning performance, mean square error was used. The mean square error of the positioning can be defined as:

(25) $$X_{rmse}=\sqrt{\frac{1}{N}\sum_{i=1}^{N}{\left(x_{i}-x_{0}\right)}^{2}}$$

(26) $$Y_{rmse}=\sqrt{\frac{1}{N}\sum_{i=1}^{N}{\left(y_{i}-y_{0}\right)}^{2}}$$

(27) $$Z_{rmse}=\sqrt{\frac{1}{N}\sum_{i=1}^{N}{\left(z_{i}-z_{0}\right)}^{2}}$$

where $X_{rmse}$is the positioning mean square errors in the X axis, $Y_{rmse}$ is the positioning mean square errors in the Y axis, $Z_{rmse}$ is the positioning mean square errors in the Z axis, and N is the number of positionings. $\left(x_{0},y_{0},z_{0}\right)$ is the true coordinate point. $\left(x_{i},y_{i},z_{i}\right)$ is the ith positioning result. The mean square error of positioning in Figure 16 is shown in Table 1.

Without spoofing, the two spoofing suppression algorithms had little impact on navigation and positioning. The algorithm of the spoofing suppression algorithm based on DOA had almost no effect on navigation and positioning. The adaptive spoofing suppression algorithm had an impact on navigation and positioning in the X, Y, Z directions of ≤0.2 m.

With spoofing, the general software receiver without spoofing suppression could not locate signals correctly; therefore, Figure 17 shows the positioning error only after suppression using the two algorithms.

Figure 17 shows the static positioning errors after using the two spoofing suppression algorithms. The receiver could locate signals correctly, and the positioning error did not increase significantly. The mean square error of positioning processed by the two algorithms is shown in Table 2.

With spoofing, the positioning performance of the spoofing suppression algorithm based on DOA was slightly better than the adaptive spoofing suppression algorithm. The adaptive spoofing suppression algorithm was better than 2.5 m in the horizontal direction and better than 4.5 m in the elevation direction. In the static test, although the positioning performance of the adaptive spoofing suppression algorithm was slightly worse than the spoofing suppression algorithm based on DOA, the calculation and implementation complexity of the adaptive spoofing suppression algorithm were much lower.

### 3.3. Kinematic Test Results

**Figure 18:**
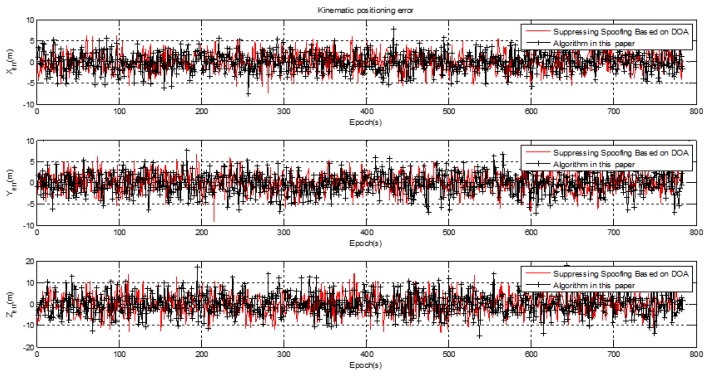
Kinematic positioning error with spoofing.

Figure 5 shows the kinematic test on a curve trajectory during human walking with a receiver from point (530524.69 m, 4235054.23 m, 95.86 m) to point (530579.18 m, 4235094.16 m, 104.10 m), which is shown by the red line in Figure 5. Post real-time kinematic (RTK) with the BeiDou B3 frequency was adopted to assess the positioning performance. The accuracy of the post-processing RTK was better than 5 cm, which meets the needs of dynamic positioning performance comparison. The positioning error for spoofing after suppression using the two algorithms is shown in Figure 18.

The kinematic positioning error had no obvious change compared with the static positioning error, which indicates that the adaptive spoofing suppression algorithm was not sensitive to the carrier motion state. The kinematic positioning mean square error is shown in Table 3.

The kinematic positioning error in the X, Y, Z axis direction was (2.38 m, 2.27 m, 4.73 m) for the spoofing suppression algorithm based on DOA, and (2.27 m, 2.43 m, 4.64 m) for the adaptive spoofing suppression algorithm. The positioning performance of the spoofing suppression algorithm based on DOA had no obvious advantage over the adaptive spoofing suppression algorithm. Compared with the static positioning performance, the positioning accuracy was almost unchanged in the horizontal direction, and the positioning accuracy was slightly worse in the elevation direction than in the static. This was caused by the fact that the dilution of precision (DOP) value of the navigation satellite in the elevation direction was worse than in the horizontal direction, and had nothing to do with the algorithm itself.

## 4. Conclusions

The adaptive spoofing suppression algorithm for GNSS based on a multiple antenna array was been proposed that could utilize the cross-correlation gain of the multiple antenna array to adaptively generate nulling and achieve the simultaneous suppression of multiple spoofing signals. The performance of the proposed algorithm was verified through static and dynamic tests. The results show that: (1) ASSA has a better suppression performance on spoofing, and can form nulls in the direction of spoofing; (2) ASSA has a small impact on navigation and positioning, with positioning errors caused by ASSA less than 0.2 m; (3) after spoofing suppression, the static positioning error in the X, Y, Z axis was (2.22 m, 2.41 m, 4.43 m) for the ASSA, which is worse than the spoofing suppression algorithm based on DOA, but the calculation and implementation complexity of the ASSA were much lower; (4) after spoofing suppression, the kinematic positioning error in the X, Y, Z axis direction was (2.27 m, 2.43 m, 4.64 m) for the ASSA, with a performance similar to the spoofing suppression algorithm based on DOA. The algorithm proposed in this paper is not sensitive to the movement state of the carrier, and has good positioning performance under both stationary and moving conditions. In the future, this algorithm will be applied to navigation receiving terminals to improve their spoofing countermeasure performances.

## Figures and Tables

**Table 1:** Static positioning performance without spoofing.

Parameter		No Spoofing	
	No Spoofing Suppression Algorithm	Suppressing Spoofing Based on DOA	Adaptive Spoofing Suppression Algorithm(ASSA)
$X_{rmse}$(m)	1.90	1.92	2.20
$Y_{rmse}$(m)	1.95	1.95	2.13
$Z_{rmse}$(m)	3.93	3.96	4.11

**Table 2:** Static positioning performance with spoofing.

Parameter		Spoofing	
	No Spoofing Suppression Algorithm	Suppressing Spoofing Based on DOA	Adaptive Spoofing Suppression Algorithm(ASSA)
$X_{rmse}$(m)	×	2.14	2.22
$Y_{rmse}$(m)	×	2.29	2.41
$Z_{rmse}$(m)	×	4.32	4.43

**Table 3:** Kinematic positioning performance with spoofing.

Parameter		Spoofing	
	No Spoofing Suppression Algorithm	Suppressing Spoofing Based on DOA Algorithm	Adaptive Spoofing Suppression Algorithm(ASSA)
$X_{rmse}$(m)	×	2.38	2.27
$Y_{rmse}$(m)	×	2.27	2.43
$Z_{rmse}$(m)	×	4.73	4.64
